# Cumulus cell DNA damage linked to fertilization success in females with an ovulatory dysfunction phenotype

**DOI:** 10.3389/fcell.2024.1448733

**Published:** 2024-11-13

**Authors:** Bárbara Rodrigues, Vanessa Sousa, Filipa Esteves, Emídio Vale-Fernandes, Solange Costa, Daniela Sousa, Raquel Brandão, Carla Leal, Joana Pires, Isabel Gaivão, João Paulo Teixeira, António J. A. Nogueira, Paula Jorge

**Affiliations:** ^1^ Molecular Genetics Laboratory, Laboratory Genetics Service, Genetics and Pathology Clinic, Centro Hospitalar Universitário de Santo António, Unidade Local de Saúde de Santo António, Porto, Portugal; ^2^ UMIB - Unit for Multidisciplinary Research in Biomedicine, ICBAS - School of Medicine and Biomedical Sciences, UPorto - University of Porto, Porto, Portugal; ^3^ ITR - Laboratory for Integrative and Translational Research in Population Health, Porto, Portugal; ^4^ Environmental Health Department, National Institute of Health Dr Ricardo Jorge, Porto, Portugal; ^5^ EPIUnit - Instituto de Saúde Pública da Universidade do Porto, Porto, Portugal; ^6^ Centre for Medically Assisted Procreation/ Public Gamete Bank, Gynaecology Departament, Centro Materno-Infantil do Norte Dr. Albino Aroso (CMIN), Centro Hospitalar Universitário de Santo António, Unidade Local de Saúde de Santo António, Porto, Portugal; ^7^ CECAV - Veterinary and Animal Research Centre and Department of Genetics and Biotechnology, University of Trás-os-Montes and Alto Douro (UTAD), Vila Real, Portugal; ^8^ CESAM – Centre for Environmental and Marine Studies, Department of Biology, University of Aveiro, Aveiro, Portugal

**Keywords:** cumulus cells, DNA damage, comet assay, ovulatory dysfunction, fertilization success, two pronuclei

## Abstract

Intracytoplasmic sperm injection (ICSI) is a widely used technique in fertility centers. ICSI success depends on both nuclear and cytoplasmic oocyte maturation. Cumulus cells, which surround the oocytes, play a pivotal role in oocyte competence. However, the significance of DNA damage in cumulus cells as a marker of fertilization success remains largely unexplored. This study aims to investigate the relationship between DNA damage in cumulus cells of females undergoing ICSI, and oocyte competence, with a focus on *in vitro* fertilization (IVF) outcomes. We employed the alkaline comet assay to assess DNA damage levels (%TDNA) in cumulus cells and whole blood from 22 potentially fertile females and 35 infertile females, including 20 with an ovulatory disfunction phenotype. Our results revealed significant differences between the levels of %TDNA in cumulus cells and blood. Females with an ovulatory dysfunction phenotype exhibited higher levels of %TDNA in cumulus cells compared to potentially fertile females. Additionally, within the group of females with ovulatory dysfunction, a significant correlation was observed between %TDNA levels and the number of oocytes with two pronuclei. Our findings suggest that blood does not accurately reflect DNA damage in cumulus cells, which was correlated with the fertilization success in females with ovulatory dysfunction. High levels of %TDNA in cumulus cells were associated with a higher likelihood of successful fertilization. Moreover, our results imply that low levels of %TDNA may be linked to oocytes that fail to complete maturation and, consequently, do not fertilize (oocytes with zero pronuclei). Further research with larger cohorts is necessary to validate these findings and to explore potential applications in female fertility. However, our study provides evidence that DNA damage in cumulus cells may serve as a valuable biomarker for predicting fertilization success and oocyte competence.

## 1 Introduction

Infertility is a complex disease characterized by the inability to achieve a clinical pregnancy after 12 months or more of regular unprotected sexual activity (Gurunath et al., 2011; Zegers-Hochschild et al., 2017; Vander Borght and Wyns, 2018). This disease affects a significant number of reproductive-aged couples globally, leading to increased reliance on assisted reproductive technologies (ART) (Nelson et al., 2013; Ombelet, 2020). Intracytoplasmic sperm injection (ICSI) is a widely utilized technique in fertility centers, with a fertilization rate between 50% and 80% (Nerl et al., 2013; Rubino et al., 2016). Nevertheless, the success rate of ICSI can vary depending on the underlying cause of infertility (Song et al., 2021). In clinical practice, the oocytes for ICSI are selected based on their nuclear maturation status, cytoplasmic structure and extracytoplasmic morphology (Rienzi et al., 2005; Lemseffer et al., 2022). The presence of the first polar body serves as a biomarker of nuclear maturity, indicating that the oocyte is at metaphase II (MII) (Veeck, 1985; Veeck, 1988; Rienzi et al., 2005; Rubino et al., 2016; Holubcová et al., 2019; Sun and Yeh, 2021; Trebichalská et al., 2021). The success of ICSI depends on both the nuclear and cytoplasmic maturity of the oocyte, which are essential for the development of pronuclei and the subsequent completion of fertilization (Eppig et al., 1996; Mobarak et al., 2019). It is crucial that the process of nuclear and cytoplasmic maturation are coordinated to establish optimal conditions for successful fertilization (Swain and Pool, 2008; Rienzi et al., 2012).

The cumulus-oocyte complex (COC), composed of an oocyte surrounded by cumulus cells, is essential for oocyte development and maturation. Cumulus cells provide a supportive microenvironment, facilitating the transfer of vital signals and nutrients via gap junctions. Additionally, these cells contribute significantly to glycolytic activity, serving as the primary energy source for oocyte maturation (Turathum et al., 2021; Martinez et al., 2023). This bi-directional communication is essential for maintaining oocyte quality (Roberts et al., 2002; Barcena et al., 2015; Turathum et al., 2021; Baratas et al., 2022). Cumulus cells play a pivotal role in COC expansion, driven by the secretion of hyaluronic acid, a process essential for meiotic maturation, ovulation, and fertilization. Furthermore, these cells support fertilization by releasing factors like prostaglandins, which enhance sperm motility and the acrosome reaction. Cumulus cells also contribute to early embryonic development, aiding in cleavage and blastocyst formation (Turathum et al., 2021; Martinez et al., 2023). At the conclusion of oocyte maturation and COC expansion, gap junctions between the oocyte and cumulus cells gradually close, leading to a loss of cell-to-cell connections (Luciano et al., 2004; Turathum et al., 2021). As a result, the cumulus cells, undergo apoptosis, characterized by DNA fragmentation (Di Giacomo et al., 2016; Majtnerová and Roušar, 2018). The close proximity between cumulus cells and oocyte suggests their potential as a biomarker for predicting oocyte competence. While studies in male infertility have linked sperm DNA damage to reproductive failure and lower fertilization rates (Simon et al., 2019; Agarwal et al., 2020; Haddock et al., 2021), research in female infertility regarding the use of cumulus cells as a biomarker for fertilization success has yield inconsistent findings (Høst et al., 2000; Abu-Hassan et al., 2006; Baratas et al., 2022). For instance, Raman et al. (2001) found a link between the comet tail moment and the percentage of fertilization following ICSI. In contrast, Tola et al. (2018) demonstrated that DNA damage in cumulus cells is not associated with oocyte and embryo quality or ICSI success. These contradictory results further underscore the importance of elucidating the relationship between cumulus cell DNA damage and oocyte competence.

The primary objective of this study was to investigate the correlation between DNA damage in cumulus cells and oocyte competence in the *in vitro* fertilization (IVF) outcomes. To complement this analysis, we investigated blood samples as a surrogate marker for DNA damage in cumulus cells, aiming to establish a reliable and accessible method for assessing DNA damage in a broader population. Cumulus cells and whole blood were collected from infertile females and those with male factor-related infertility undergoing ICSI. DNA damage was quantified using the alkaline comet assay, and comparisons were made between sets with different fertility status, to explore correlations between DNA damage levels, ovarian reserve markers and with IVF outcomes.

## 2 Materials and methods

### 2.1 Study design and population

This study investigated the outcomes of ICSI in non-smoking females (n = 57). Two sets were defined based on the cause of infertility: set 1 (potentially fertile), consisted of 22 females experiencing male factor-related infertility, obtained by convenience sampling; set 2, comprised 35 infertile females, arbitrarily selected using Random Sequence Generator (https://www.random.org/, accessed on 05 November 2022) from a larger cohort. This cohort included females diagnosed with various female factor infertility etiologies: ovulatory dysfunction (n = 83, 66.9%), endometriosis (n = 17, 13.7%), and oocyte factor (n = 12, 9.7%). Less frequent diagnoses (n = 12, 9.7%) included hypothyroidism, hyperprolactinemia, diminished ovarian reserve, adenomyosis, obesity, among others. Age-range were matched between the two groups to control for potential confounding effects of age on hormonal secretory patterns (van den Beld et al., 2018).

Participants were recruited between mid-January/2020, and February/2022 from the Centre for Medically Assisted Procreation of the Centro Materno-Infantil do Norte Dr. Albino Aroso (CMIN-ULSSA). Recruitment was temporarily paused between March and November/2020 due to COVID-19 pandemic. The study was approved by the Ethics Committee of the Unidade Local de Saúde de Santo António (ULSSA) (process number 2020.119/097-DEFI/099-CE). Prior to enrollment, all participants provided written informed consent after receiving detailed information about the study’s objectives, procedures, potential risks, and benefits. Participants were given the opportunity to ask questions and ensure their understanding before signing the consent form. It was emphasized that participation would not affect their medical treatment, and that collected data would be used exclusively for research purposes. Consent was obtained during the initial phase of ovarian stimulation to align with the timing of sample collection.

### 2.2 Fertility-related outcomes and demographic variables

Baseline demographic data, including age and infertility etiology were collected from all participants. Ovarian reserve markers were assessed, encompassing day 3 follicle-stimulating hormone [FSH], anti-Müllerian hormone [AMH] within 6 months prior to ovarian stimulation, and antral follicle count [AFC] (Table 1). Additionally, IVF outcomes were captured, including ovarian stimulation response (total gonadotrophin dose, stimulation duration, follicle count on the trigger day, and oocyte retrieval), oocyte quality (mature MII oocytes), and fertilization success (pronuclear configuration: 0 PN, 1 PN, 2 PN, 3 PN) (Table 2).

**TABLE 1:** Descriptive analysis of the ovarian reserve markers according to fertility status.

		Set 1		Set 2	*p*-value^a^
	n	Median	n	Median	
		(Q1, Q3)		(Q1, Q3)	
Age (years)	22	33.5 (31.0, 35.5)	35	35.0 (32.0, 38.0)	0.103^b^
Ovarian reserve markers					
Day 3 FSH (mUI/mL)	22	7.8 (6.2, 9.6)	33	6.7 (5.1, 8.6)	0.087
AMH (ng/mL)	17	8.5 (5.5, 3.1)	33	7.8 (4.6, 15.0)	0.822
AFC	16	8.0 (6.0, 11.5)	23	7.0 (6.0, 9.0)	0.687

Values are presented as median (Quartile 1, Quartile 3); Set 1: Females with male factor-related infertility; Set 2: Infertile females; FSH: Follicle-stimulating hormone; AMH: Anti-Müllerian hormone; AFC: Antral follicle count.

^a^ *p*-value of Mann-Whitney test.

^b^ *p*-value of *t*-test.

**TABLE 2:** Descriptive analysis of the IVF outcomes according to fertility status.

		Set 1		Set 2	*p*-value^a^
	n	Median	n	Median	
		(Q1, Q3)		(Q1, Q3)	
Response to ovarian stimulation					
Number of follicles on the trigger day	22	7.5 (4.8, 11.5)	35	6.0 (2.0, 11.0)	0.289
Number of oocytes retrieved	22	11.5 (8.8, 16.5)	35	10.0 (6.0, 16.0)	0.367
Oocyte maturation					
Number of injected MII oocytes	22	9.0 (4.0, 12.5)	35	7.0 (4.0, 11.0)	0.267
Fertilization success					
Number of oocytes with 0 PN	22	2.0 (0, 4.3)	34	1.0 (1.0, 3.0)	0.427
Number of oocytes with 1 PN	22	0 (0, 1.0)	34	0 (0, 0)	0.257
Number of oocytes with 2 PN	22	5.0 (3.0, 9.0)	34	5.0 (2.0, 7.0)	0.506
Number of oocytes with 3 PN	22	0 (0, 0.3)	34	0 (0, 0)	0.059

	n	Mean ± s.d	n	Mean ± s.d	*p*-value^b^
Therapeutic regime					
Total dose of gonadotrophins (IU/mL)	22	2,519.3 ± 766.9	35	2,684.3 ± 706.9	0.205
Stimulation duration (days)	22	10.7 ± 1.6	35	10.5 ± 1.9	0.288

Values are presented as median (Quartile 1, Quartile 3) for non-parametric data and mean ± standard deviation (s.d.) for normally distributed data; Set 1: Females with male factor-related infertility; Set 2: Infertile females; 0 PN: Zero pronuclei; 1 PN: One pronuclei; 2 PN: Two pronuclei; 3 PN: Three pronuclei.

^a^ *p*-value of Mann-Whitney test.

^b^ *p*-value of *t*-test.

### 2.3 Alkaline comet assay

#### 2.3.1 Collection of whole blood and cumulus cells

EDTA K3 tubes (VACUETTE^®^, Greiner AG, Kremsmünster, Austria) were used to collect whole blood samples from each female on the day of follicular puncture. The samples were immediately stored at −70 °C until further use. Cumulus cells were also obtained on the same day, following oocyte denudation using enzymatic (ICSI Cumulase^®^ enzyme [Origio^®^, Måløv, Denmark]) and mechanical methods. Cells from all oocytes retrieved per female were pooled and cryopreserved in liquid nitrogen. All samples were anonymized to ensure confidentiality.

#### 2.3.2 Positive and negative controls

Negative and positive controls were established using a pool of blood samples obtained from five non-smoking female volunteers within a comparable age range as the study participants. The positive control was exposed to a concentration of 5 mM methyl methanesulfonate (Merck KGaA, Darmstadt, Germany) for a duration of 1 hour at 37 °C. Following this treatment, the cells were cryopreserved in liquid nitrogen.

#### 2.3.3 Assessment of DNA damage

The alkaline comet assay was performed, following minor modifications in the protocol based on the original methods described by Singh et al. (1988), subsequently adapted by Abreu et al. (2017) and Collins et al. (2023). Briefly, thawed cumulus cell pellets at 37 °C, were washed with PBS (Sigma-Aldrich^®^), and resuspended in 0.8% low-melting point agarose (LMA) (Sigma-Aldrich^®^). Thawed whole blood samples on ice at room temperature were directly resuspended in 0.8% LMA. Two-gel slides were prepared for each sample by dropping 70 µL of cell suspension into each gel, on a frosted slide precoated with 1% normal melting point agarose (GRS Agarose LE, Grisp, Porto, Portugal) and covered with 20 × 20 coverslips. Slides were placed at 4°C for 5 min to solidify. Coverslips were removed, and slides were immersed in a freshly prepared lysis solution (2.5 M NaCl, 0.1 M EDTA, 0.01 M Tris, 10 M NaOH, and 1% Triton X-100, pH = 10) at 4 °C for 90 min in the dark. Slides were placed in an electrophoresis tank with cold buffer (0.3 M NaOH and 0.01 M EDTA, pH = 13) at 4 °C for 30 min, to allow DNA unwinding. Electrophoresis was carried out at 4 °C for 20 min at 25 V (1v/cm). Slides were washed with neutralizing buffer (PBS, Sigma-Aldrich^®^), at 4 °C for 10 min, dehydrated/fixed with ethanol 96% (10 min) (Merck KGaA, Frankfurter, Darmstadt, Germany), and let to dry overnight. Dried slides were stained with SYBR™ Gold (Invitrogen™, Waltham, MA, United States) at room temperature for 30 min. Analyses were performed using Motic BA410E epi-fluorescence microscope (Motic^®^, Barcelona, Spain), with 400x magnification, and Comet Assay IV (Instem^®^, Staffordshire, UK) software. A total of 150 cells (75 per gel) were scored per sample type (cumulus and blood cells) for each female.

According to Organization for Economic Co-operation and Development (OECD) guidelines for *in vivo mammalian* alkaline comet assay (test no. 489), the percentage of DNA in the comet tail (%TDNA) was used to evaluate DNA damage at cell level on a scale from 0% to 100% (OECD/OCDE, 2016).

### 2.4 Statistical analysis

The minimum sample size (n = 33) was estimated based on Pearson’s correlation coefficient, assuming an expected correlation of 0.5, a 95% confidence level, and a statistical power of 80% (Bonett and Wright, 2000). To compare fertility-related outcomes (total dose of gonadotrophins and stimulation duration), *t*-test were conducted. When the data failed normality and homoscedasticity, Mann-Whitney test were employed (FSH, AMH, AFC, number of follicles on the trigger day, number oocytes retrieved, number of injected MII oocytes, number of 0 PN, 1 PN, 2 PN, 3 PN oocytes and %TDNA levels). Descriptive statistics for variables that did not follow a normal distribution were reported as median with interquartile range (quartile 1, quartile 3), while mean ± standard deviation (s.d.) was used for normally distributed data. A two-way analysis of variance (ANOVA) was employed to evaluate differences in DNA damage levels (log-%TDNA), considering fertility status and type of cells as independent variables. The study adhered to a fully randomized factorial design, ensuring independent and randomly selected datasets. To account for potential interactions between explanatory variables, a factorial ANOVA model was utilized. Following two-way ANOVA, Dunnett’s multiple comparison test was conducted. Pearson correlation coefficient was used to assess the linear correlation (linear association) between log-%TDNA and fertility-related outcomes (FSH, log-AMH, AFC, total dose of gonadotrophins, stimulation duration number of follicles on the trigger day, number of oocytes retrieved, number of injected MII oocytes, square-root of the number of oocytes with 0 PN and square-root of the number of oocytes with 2 PN). All statistical analyses were performed using SigmaPlot version 14.0 (Systat Software^®^ Inc., Chicago, IL, United States). A statistical significance level of 0.05 was used for all tests.

## 3 Results

### 3.1 Demographic and clinical characteristics

This study included 22 females with male-related infertility with a mean age of 33.6 ± 3.2 years, ranging from 28 to 39 years and 35 infertile females a mean age of 34.8 ± 3.5 years, ranging from 25 to 39 years. The primary causes of infertility were ovulatory dysfunction (n = 20, 57.1%), oocyte factor (n = 6, 17.1%), and endometriosis (n = 5, 14.3%). Additional causes, including hypothyroidism, hyperprolactinemia, diminished ovarian reserve, and adenomyosis were less prevalent (n = 4, 11.5%). Tables 1, 2 summarize the demographic and clinical characteristics of the two sets. Consistent with previous reports, the majority of participants exhibited an ovulatory dysfunction phenotype, characterized by irregular or absent menstruation and anovulation (Carson and Kallen, 2021; Munro et al., 2023).

### 3.2 Infertile and potentially fertile females showed similar conventional fertility-related outcomes

Ovarian reserve markers, including FSH, AMH, and AFC, were assessed in both sets (Table 1). Despite matching for age, no significant differences were found in ovarian reserve markers between infertile and potentially fertile females (Table 1). Furthermore, IVF outcomes, including total dose of gonadotrophins, stimulation duration, number of follicles on the trigger day, number of oocytes retrieved and MII oocytes injected were comparable between the two sets. Subsequently, we evaluated the fertilization rates by categorizing the oocytes into non-fertilized (0 PN), abnormally fertilized (1 PN and 3 PN), and normally fertilized (2 PN). No statistically significant differences were found in the number of oocytes with 0 PN, 1 PN, 3 PN as well as 2 PN (Table 2). These findings suggest that infertile and potentially fertile females may exhibit similar ovarian reserve characteristics and IVF outcomes, highlighting the complexity of infertility and the potential for successful outcomes in both sets.

### 3.3 Distinct levels of DNA damage in blood and cumulus cells

**FIGURE 1:**
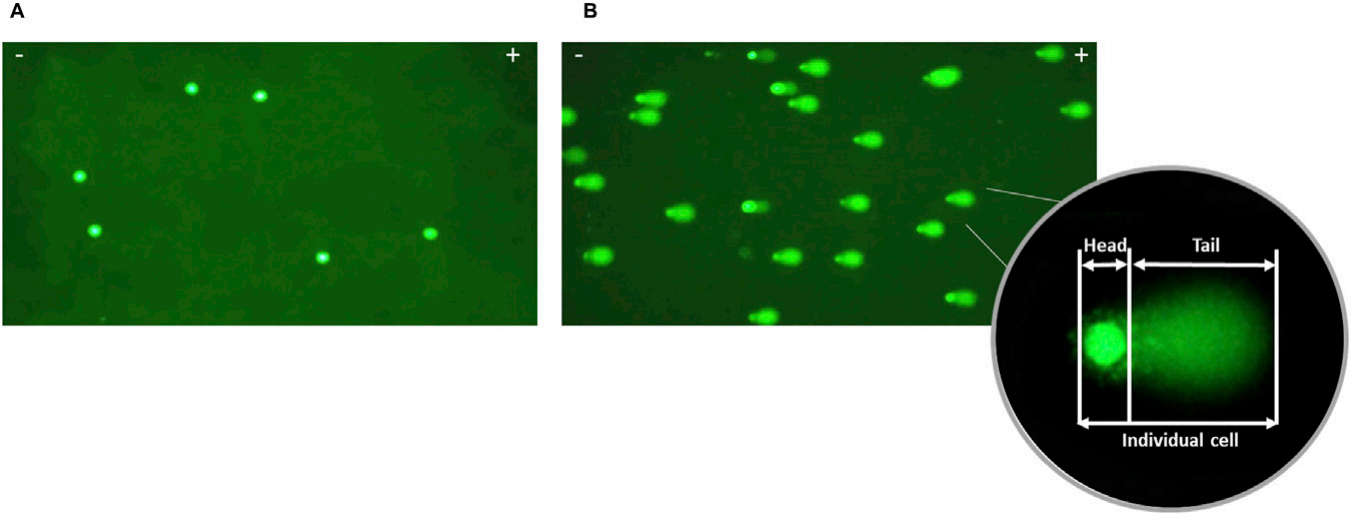
Visualization of DNA damage in cumulus cells after alkaline comet assay. **(A)** Negative control: intact nuclei without DNA damage (%TDNA = 1.16); **(B)** Positive control: exposure to MMS resulted in significant high DNA damage and insignificant head (%TDNA = 49.66); Tail intensity represents the percentage of DNA fragments the comet’s tail, indicating the extent of DNA damage; - cathode; + anode.

To evaluate the extent of DNA damage in blood and cumulus cells, the comet assay was simultaneously conducted on control samples. Cells with no visible DNA in the tail (negative control, Figure 1A) served as a baseline, while those with DNA in the tail (positive control, Figure 1B) indicated DNA damage. A variation coefficient of 15% and 20%, was observed in the positive and negative controls, respectively, ensuring the assay’s reproducibility (Collins et al., 2014).

**FIGURE 2:**
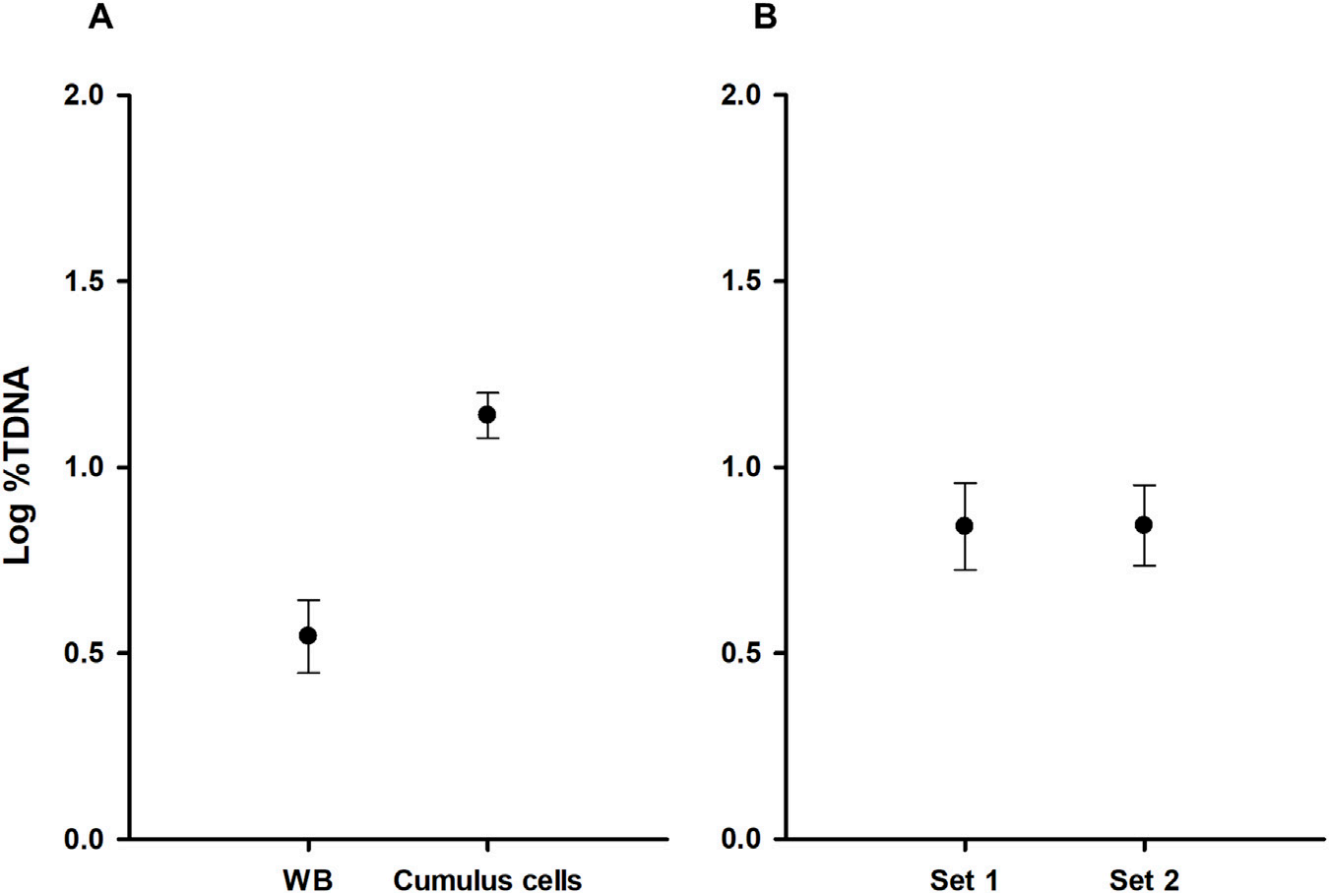
Comparative analysis of DNA damage levels according to sets and tissues. **(A)** Comparison of DNA damage levels in whole blood (WB) and cumulus cells; **(B)** Comparison between set 1 (females with male factor-related infertility) and set 2 (infertile females); %TDNA–levels of DNA damage.

**FIGURE 3:**
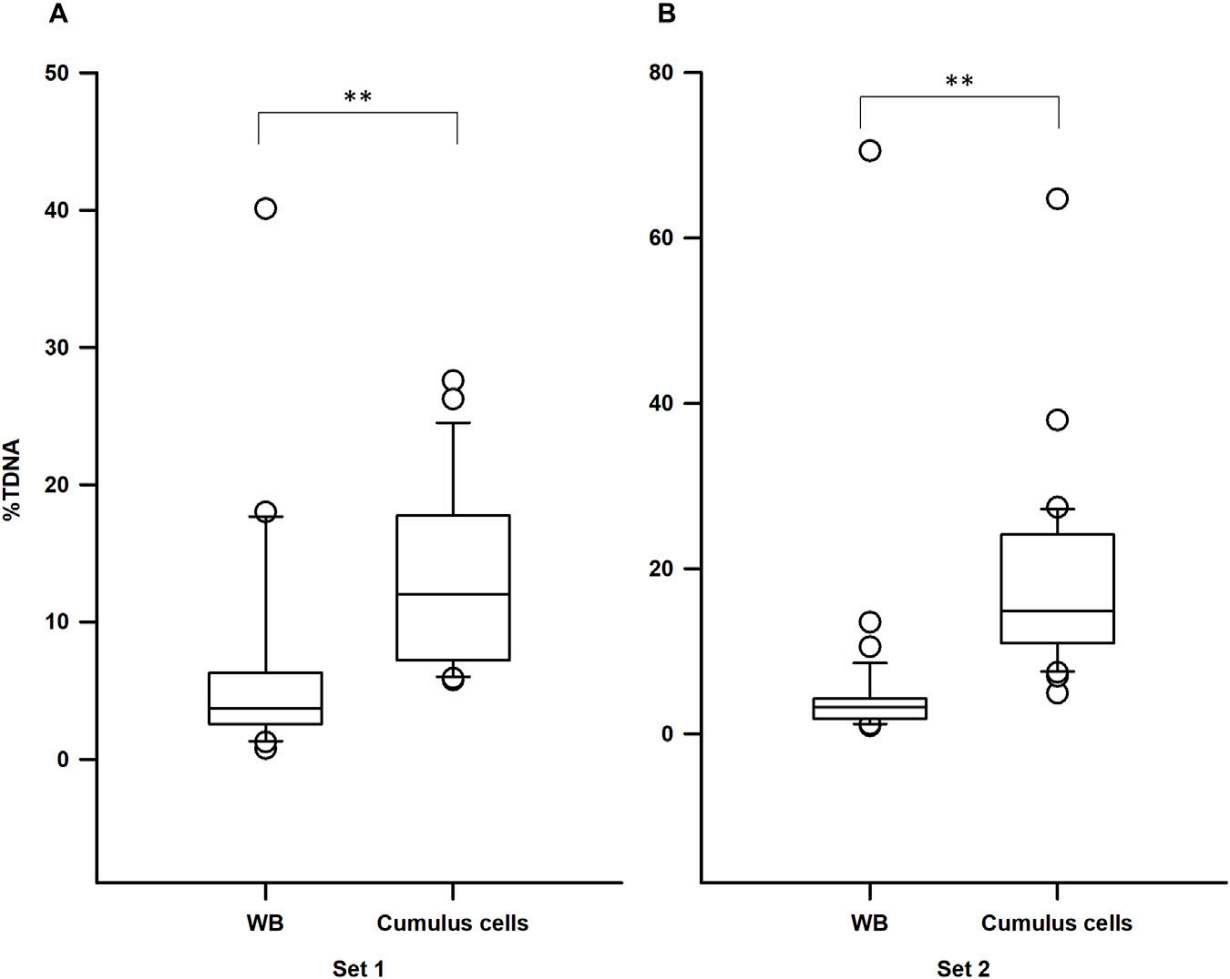
Tissue-specific differences in DNA damage levels in each set. **(A)** Set 1 (females with male factor-related infertility): cumulus cells exhibited significantly higher DNA damage levels compared to whole blood (WB); **(B)** Set 2 (infertile females): similarly, to set 1, cumulus cells displayed significantly elevated DNA damage levels compared to WB; %TDNA–DNA damage levels; **p < 0.001, calculated using the Mann-Whitney test.

An association analysis of DNA damage levels (%TDNA) in blood and cumulus cells from the same individuals revealed no significant correlation (Pearson correlation: r = −0.095, n = 57, *p* = 0.481). To investigate the influence of tissue type and fertility status on DNA damage a two-way ANOVA was performed. Log-%TDNA values exhibited normality (Shapiro-Wilk test: W = 0.984; *p* = 0.189) and equal variance (Brown-Forsythe test: *p* = 0.182). The ANOVA results demonstrated a significant effect of tissue type on log-%TDNA (F _(1, 110)_ = 93.286, *p* < 0.001) (Figure 2A), but no interaction between tissue type and fertility status (F _(1, 110)_ = 3.832, *p* = 0.053), nor a significant effect of fertility status (F _(1, 55)_ = 0.002, *p* = 0.964) (Figure 2B). These findings suggest that while tissue type significantly influences DNA damage, fertility status does not. Similar results were observed when analyzing %TDNA levels according to the fertility status (Figure 3). Supplementary Table S1 provides detailed statistical analyses for each sample.

**FIGURE 4:**
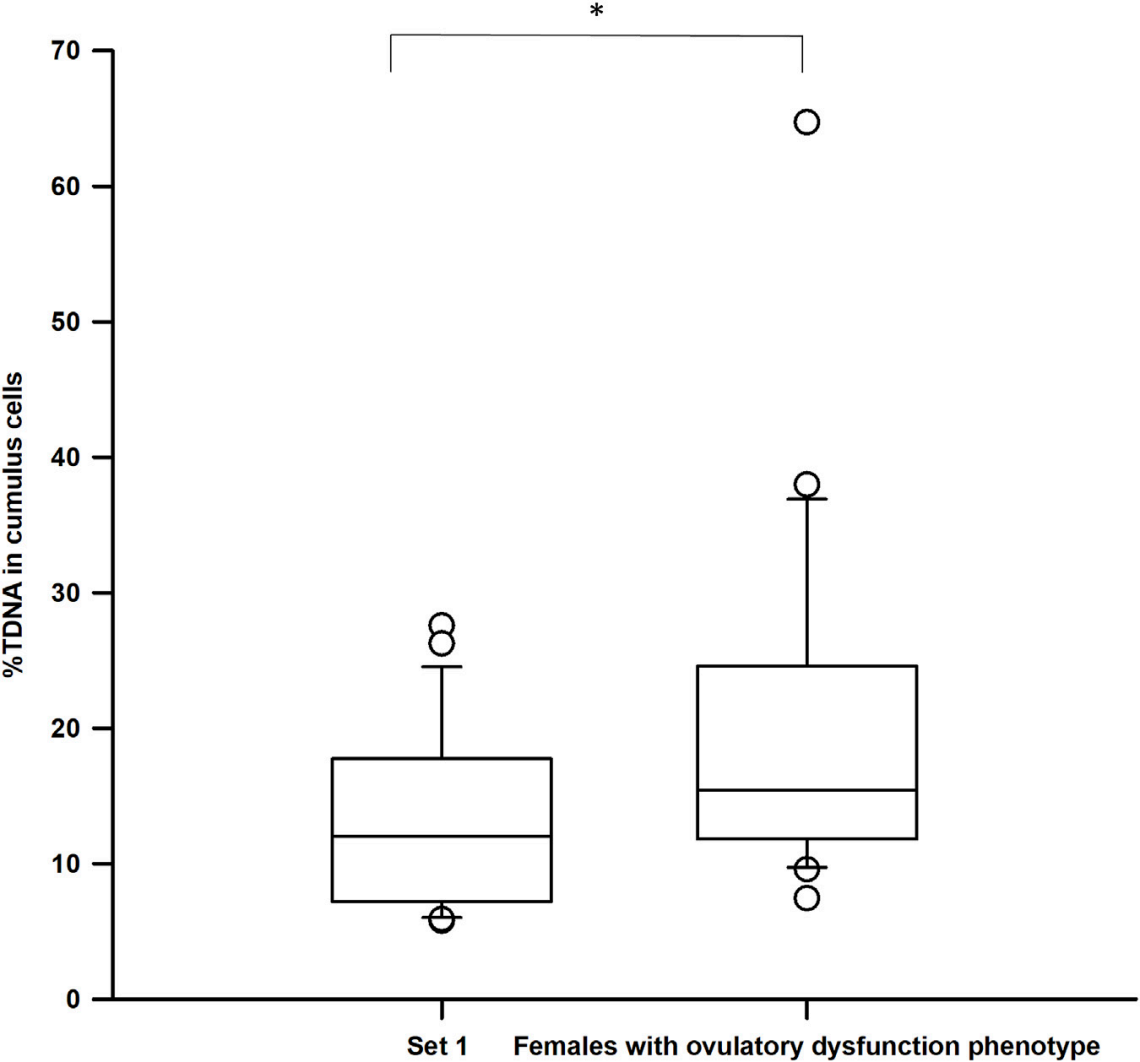
Comparison of DNA damage levels between set 1 (females with male factor-related infertility) and infertile females with ovulatory dysfunction; %TDNA–DNA damage levels; *p < 0.05, calculated using the Mann-Whitney test.

A Mann-Whitney test revealed no statistically significant differences in %TDNA levels between cumulus cells from infertile and potentially fertile females (U = 274.5; n_1_ = 22; n_2_ = 35; *p* = 0.07). Given this lack of statistical significance, we hypothesized that heterogeneity of infertility causes might be influencing these outcomes. When analyzing only infertile females with ovulatory dysfunction (20 out of 35 cases) we observed significant higher %TDNA levels in this group compared to potentially fertile females (Mann-Whitney test: U = 135.5; n_1_ = 20; n_2_ = 22; *p* = 0.034), as depicted in Figure 4. Importantly, no significant differences were observed in fertility-related outcomes between these two sets (*p* > 0.05 all variables).

### 3.4 Impact of cumulus cell DNA damage in females with an ovulatory dysfunction phenotype

**FIGURE 5:**
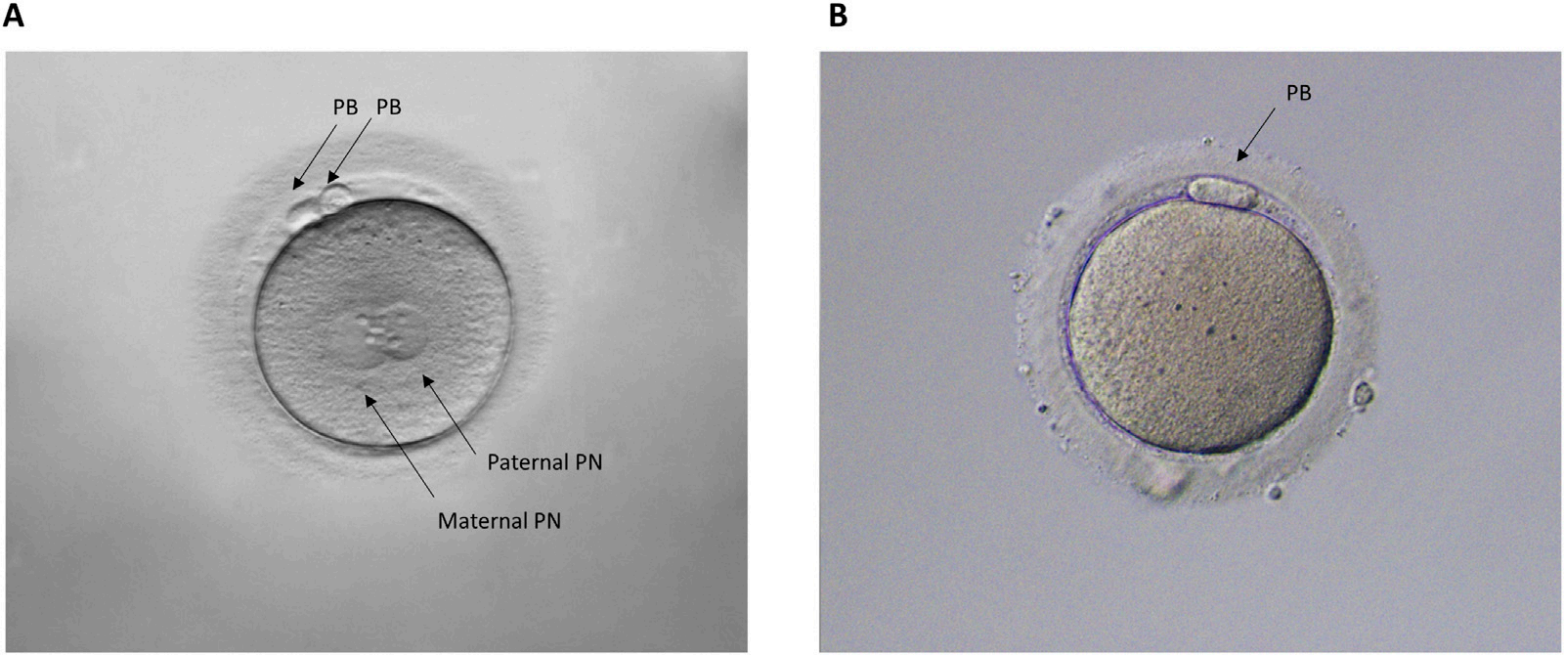
Representative human MII oocytes. **(A)** Normal fertilization: oocyte with two pronuclei (2 PN, maternal and paternal) and two polar bodies; **(B)** Unfertilized oocyte: zero pronuclei (0 PN) and a single polar body (PB).

To investigate the association between DNA damage in cumulus cells and ovarian function and competence, we examined the correlation between log-%TDNA levels and ovarian reserve markers (FSH, log-AMH and AFC) and IVF outcomes. Pearson correlation analysis revealed no significant correlations between log-%TDNA and the ovarian reserve markers (Table 3). Additionally, no correlations were observed between log-%DNA and IVF outcomes, including total dose of gonadotrophins, stimulation duration, number of follicles on the trigger day, number of oocytes retrieved, number of injected MII oocytes and square-root of number of 0 PN oocytes) (Table 4). A significant positive correlation was found between log-%TDNA and the square-root of the number of 2 PN oocytes (Pearson correlation: r = 0.508, n = 19, *p* = 0.026), indicating an association with fertilization success (Table 4; Figure 5A). In addition, infertile females with ovulatory dysfunction and a high number of 0 PN oocytes (≥8) exhibited lower levels of %TDNA levels in cumulus cells compared to those with fewer 0 PN oocytes (Figure 5B; Supplementary Table S2). Although this result did not reach statistical significance (Mann-Whitney test: U = 14.0; n_1_ = 3; n_2_ = 16; *p* = 0.288), it suggests a potential association between reduced %TDNA levels and oocyte competence, particularly in cases with a high number of 0 PN oocytes.

**TABLE 3:** Summary of correlation analysis with ovarian reserve markers in females with ovulatory dysfunction.

	Correlation coefficients with levels of DNA damage in cumulus cells^a^	*p*-value	n
Markers of ovarian reserve			
Day 3 FSH (mUI/mL)	−0.247	0.294	20
AMH (ng/mL)^a^	−0.112	0.639	20
AFC	0.153	0.674	10

^a^ Log-transformed data; FSH: Follicle-stimulating hormone; AMH: Anti-Müllerian hormone; AFC: Antral follicle count.

**TABLE 4:** Summary of correlation analysis with IVF outcomes in females with ovulatory dysfunction.

	Correlation coefficients with levels of DNA damage in cumulus cells^a^	*p*-value	n
Therapeutic regime			
Total dose of gonadotrophins (IU/mL)	0.225	0.339	20
Stimulation days	0.204	0.388	20
Response to ovarian stimulation			
Number of follicles on the trigger day	−0.053	0.825	20
Number of oocytes retrieved	0.054	0.821	20
Oocyte maturation			
Number of injected MII oocytes	0.323	0.165	20
Fertilization success			
Number of oocytes with 0 PN^b^	−0.080	0.746	19
Number of oocytes with 2 PN^b^	0.508	0.026^c^	19

^a^ Log-transformed data.

^b^ Square root-transformed data.

^c^ Significant correlation; MII: Metaphase II; 0 PN: Zero pronuclei; 2 PN: Two pronuclei.

## 4 Discussion

This study aimed to investigate the relationship between DNA damage in cumulus cells of females undergoing ICSI and oocyte competence, with a focus on IVF outcomes.

Blood samples have been considered a valuable tool for evaluating global DNA damage, for this we evaluated whether this tissue could mirror the effects of DNA damage in cumulus cells. Our findings suggest that it may not be a suitable sentinel for cumulus cell DNA damage, posing a limitation for future studies due to the inherent challenges associated in obtaining cumulus cells.

This study comprised a set of infertile females with diverse etiologies of infertility, with a predominance of ovulatory dysfunction phenotypes, consistent with previous findings (Carson and Kallen, 2021). Our results revealed a significant increase in the levels of %TDNA in cumulus cells of females with ovulatory dysfunction compared to potentially fertile females. Although the sample size was relatively small, the robustness of our conclusions was supported by strong statistical power. Notably, we observed a significant positive correlation between the levels of %TDNA in cumulus cells and the number of 2 PN oocytes, suggesting that DNA damage level in cumulus cells may indeed be relevant predictor of fertilization success and a potential indicator of oocyte competence. In alignment with our hypothesis, a sample with higher levels of %TDNA (27%) exhibited a high fertilization success rate, yielding twelve 2 PN oocytes from thirteen injected MII oocytes (sample 24, Supplementary Table S1, S2). Conversely, a sample with lower %TDNA levels (7%) demonstrated a low rate of fertilization success, resulting in only one 2 PN oocyte from ten injected MII oocytes (sample 37, Supplementary Table S1, S2). The development of pronuclei and the subsequent completion of fertilization depend on the nuclear and cytoplasmatic maturation, i.e., oocyte competence (Rienzi et al., 2012; Coticchio et al., 2014; Trebichalská et al., 2021; Liao et al., 2023). Interestingly, in sample 37, despite the injection of ten MII oocytes nine did not fertilize (nine 0 PN [zero pronuclei] oocytes), Supplementary Table S2). While other factors may have contributed, it is plausible that the 0 PN oocytes were immature, likely due to insufficient cytoplasmic maturation, leading to no fertilization. The preserved DNA integrity of cumulus cells, as indicated by lower levels of % TDNA, could potentially serve as a reliable biomarker of oocyte competence, addressing a significant knowledge gap in reproductive biology. Our findings align with those of Raman et al. (2001) who demonstrated a correlation between the comet tail moment and the percentage of fertilization following ICSI. Furthermore, Lourenço et al. (2014) observed elevated caspase activity, one of the apoptotic markers they analyzed, in cumulus cells from fertilized oocytes compared to unfertilized ones, suggesting a potential link to oocyte quality and fertilization ability. However, the literature presents conflicting evidence regarding this association (Høst et al., 2002; Corn et al., 2005). Høst et al. (2000) and Lee et al. (2001) reported results suggesting that high levels of cumulus cell apoptosis may compromise fertilization. Abu-Hassan et al. (2006) found no clear association between cumulus cell apoptosis levels and IVF outcomes. Similarly, Tola et al. (2018) demonstrated a lack of correlation between DNA integrity of cumulus cells and oocyte and embryo quality or ICSI success.

The discrepancies in these findings may be attributed to variations in study design, such as evaluation of DNA damage in pooled or individually isolated cumulus cells, sample size, and the methodologies employed to assess apoptosis, including the incidence of apoptosis, apoptotic markers, percentage of viable cells, or DNA fragmentation. Additionally, the specific fertility parameters evaluated in each study may contribute to the observed differences. The detachment and apoptosis of cumulus cells during COC expansion, as documented by Szołtys et al. (2000) and Luciano et al. (2004), underscore the critical nature of this cellular interaction. While the precise mechanisms governing the relationship between DNA damage and apoptosis during oocyte maturation remain enigmatic, further investigations with larger sample sizes and additional biological endpoints are essential to elucidate these pathways and explore potential therapeutic applications for addressing female infertility.

## 5 Conclusion

Overall, these findings suggest that DNA damage in cumulus cells may serve as a predictive marker of fertilization success. Furthermore, the study provides insights into the association between low levels of total DNA damage and the absence of cytoplasmic maturation. These results highlight the potential significance of cumulus cell DNA damage as an indicator of oocyte development and maturation. A more comprehensive understanding of the intricate relationship between cumulus cells and oocyte development could lead to the identification of valuable biomarkers for assessing oocyte quality, maturation, fertilization, embryonic development, and cumulus cell function, ultimately contributing to the optimization of routine IVF procedures.

## Data Availability

The original contributions presented in the study are included in the article/Supplementary Material, further inquiries can be directed to the corresponding author.
