# Supplementary material for: Cumulus cell DNA damage linked to fertilization success in females with an ovulatory dysfunction phenotype
Source: Front Cell Dev Biol. 2024 Nov 13;12:1448733. doi: 10.3389/fcell.2024.1448733 (PMC11599247; doi:10.3389/fcell.2024.1448733)
Supplement: Supplementary file 1 [file Table1.docx]

Supplementary Material

# Supplementary Data

# Supplementary Tables

**Supplementary Table 1.** Levels of DNA damage (%TDNA) in whole blood and cumulus cells of females from sets 1 (females with male factor-related infertility; **bold samples**) and 2 (infertile females; underlined samples).

| **Sample** | **Levels of %TDNA in whole blood** | **Levels of %TDNA in cumulus cells** |
| --- | --- | --- |
| **1** | 8.2 | 7.4 |
| **2** | 2.1 | 18.3 |
| **3** | 5.3 | 6.6 |
| **4** | 1.5 | 9.6 |
| **5** | 3.4 | 10.8 |
| **6** | 7.2 | 5.8 |
| **7** | 2.6 | 6.5 |
| **8** | 18.0 | 26.2 |
| **9** | 4.3 | 12.0 |
| **10** | 6.0 | 10.3 |
| **11** | 4.0 | 6.3 |
| **12** | 16.9 | 13.3 |
| **13** | 4.5 | 27.6 |
| **14** | 40.1 | 5.9 |
| **15** | 3.0 | 17.6 |
| **16** | 0.8 | 12.7 |
| **17** | 2.5 | 12.0 |
| **18** | 2.6 | 19.6 |
| **19** | 2.7 | 11.1 |
| **20** | 1.3 | 12.0 |
| **21** | 4.0 | 20.5 |
| **22** | 3.2 | 13.5 |
| 23 | 3.3 | 11.9 |
| 24 | 3.3 | 27.1 |
| 25 | 1.1 | 16.0 |
| 26 | 3.5 | 12.3 |
| 27 | 1.4 | 11.3 |
| 28 | 3.5 | 38.0 |
| 29 | 6.4 | 22.1 |
| 30 | 1.2 | 11.8 |
| 31 | 5.9 | 7.6 |
| 32 | 1.9 | 8.8 |
| 33 | 70.6 | 14.8 |
| 34 | 2.6 | 11.0 |
| 35 | 3.0 | 15.0 |
| 36 | 2.4 | 18.9 |
| 37 | 1.6 | 7.4 |
| 38 | 4.9 | 27.4 |
| 39 | 1.1 | 9.6 |
| 40 | 1.9 | 17.3 |
| 41 | 3.2 | 7.0 |
| 42 | 1.6 | 64.7 |
| 43 | 13.5 | 14.8 |
| 44 | 10.5 | 22.1 |
| 45 | 3.3 | 12.7 |
| 46 | 1.0 | 24.9 |
| 47 | 3.5 | 4.9 |
| 48 | 3.9 | 9.5 |
| 49 | 2.7 | 11.2 |
| 50 | 1.8 | 24.1 |
| 51 | 4.4 | 19.0 |
| 52 | 7.3 | 21.9 |
| 53 | 4.2 | 13.6 |
| 54 | 1.8 | 25.4 |
| 55 | 2.8 | 25.0 |
| 56 | 3.6 | 7.7 |
| 57 | 2.5 | 24.3 |

**Supplementary Table 2.** Fertility-related outcomes of females from sets 1 (females with male factor-related infertility; **bold samples**) and 2 (infertile females; underlined samples).

| **Sample** | **Age** | **Day 3 FSH** | **LH** | **AMH** | **AFC** | **Total dose of gonadotrophins (IU/ml)** | **Stimulation duration (days)** | **Nº of follicles on the trigger day** | **Nº of oocytes retrieved** | **Nº of injected MII oocytes** | **Nº of oocytes with 0PN** | **Nº of oocytes with 1PN** | **Nº of oocytes with 2PN** | **Nº of oocytes with 3PN** |
| --- | --- | --- | --- | --- | --- | --- | --- | --- | --- | --- | --- | --- | --- | --- |
| **1** | 34 | 6.3 | 3.6 | 2.2 | 14 | 1800 | 8 | 11 | 14 | 11 | 4 | 0 | 6 | 0 |
| **2** | 38 | 10.5 | 9.1 | 1.3 | - | 3900 | 13 | 13 | 14 | 14 | 4 | 1 | 8 | 0 |
| **3** | 29 | 4.9 | 3.6 | 10.0 | 9 | 2400 | 12 | 14 | 33 | 3 | 15 | 1 | 0 | 13 |
| **4** | 39 | 6.9 | 7.3 | 1.4 | - | 2250 | 9 | 4 | 5 | 4 | 0 | 0 | 4 | 0 |
| **5** | 32 | 8.8 | 9.2 | 2.0 | 5 | 1950 | 9 | 3 | 7 | 4 | 3 | 0 | 1 | 0 |
| **6** | 35 | 12.1 | 8.5 | 2.7 | 5 | 3300 | 11 | 7 | 11 | 11 | 2 | 0 | 9 | 0 |
| **7** | 35 | 8.8 | 4.0 | 1.3 | 10 | 2175 | 10 | 6 | 9 | 5 | 2 | 0 | 3 | 0 |
| **8** | 35 | 10.4 | 6.9 | 4.4 | 10 | 2200 | 11 | 9 | 16 | 15 | 5 | 1 | 7 | 0 |
| **9** | 33 | 5.7 | 1.6 | 3.8 | 25 | 1350 | 9 | 15 | 11 | 10 | 5 | 1 | 4 | 0 |
| **10** | 33 | 7.3 | 4.1 | 2.3 | - | 3600 | 12 | 1 | 9 | 4 | 1 | 0 | 3 | 0 |
| **11** | 35 | 8.0 | 10.5 | 2.7 | - | 1500 | 10 | 2 | 8 | 7 | 2 | 0 | 3 | 1 |
| **12** | 39 | 10.0 | 5.1 | 1.1 | 4 | 3900 | 13 | 5 | 4 | 3 | 0 | 0 | 1 | 2 |
| **13** | 30 | 6.6 | 8.9 | 5.8 | - | 2500 | 13 | 10 | 37 | 18 | 0 | 0 | 14 | 0 |
| **14** | 38 | 9.4 | 27.5 | 2.3 | 10 | 2800 | 13 | 18 | 8 | 4 | 0 | 0 | 4 | 0 |
| **15** | 31 | 13.4 | 6.0 | - | 2 | 3750 | 13 | 6 | 9 | 7 | 0 | 1 | 6 | 0 |
| **16** | 30 | 8.7 | 6.0 | 2.7 | 12 | 2250 | 10 | 1 | 13 | 12 | 5 | 0 | 6 | 0 |
| **17** | 31 | 7.6 | 9.8 | 2.8 | 6 | 2000 | 10 | 8 | 11 | 6 | 2 | 0 | 3 | 1 |
| **18** | 34 | 8.1 | 5.0 | - | 7 | 2025 | 9 | 6 | 12 | 11 | 7 | 0 | 4 | 0 |
| **19** | 37 | 5.2 | 9.8 | - | 5 | 2250 | 10 | 11 | 24 | 20 | 3 | 1 | 13 | 1 |
| **20** | 32 | 6.8 | 5.3 | - | 6 | 3300 | 11 | 7 | 12 | 8 | 0 | 0 | 6 | 0 |
| **21** | 28 | 7.4 | 8.3 | - | 16 | 2025 | 9 | 9 | 21 | 18 | 3 | 0 | 15 | 0 |
| **22** | 31 | 5.3 | 4.8 | 6.8 | - | 2200 | 11 | 17 | 18 | 12 | 0 | 0 | 12 | 0 |
| 23 | 28 | 4.4 | 13.1 | 10.9 | - | 2025 | 9 | 13 | 25 | 10 | 1 | 0 | 8 | 0 |
| 24 | 34 | 4.9 | 14.1 | 10.2 | - | 1700 | 12 | 21 | 25 | 13 | 1 | 0 | 12 | 0 |
| 25 | 39 | 4.1 | 10.4 | 3.9 | - | 1575 | 7 | 12 | 25 | 14 | 8 | 0 | 5 | 0 |
| 26 | 38 | 7.4 | 17.5 | 18.9 | - | 2250 | 10 | 14 | 32 | 13 | 3 | 0 | 9 | 0 |
| 27 | 32 | 7.0 | 14.6 | 7.0 | - | 1350 | 9 | 11 | 23 | 11 | 2 | 0 | 7 | 0 |
| 28 | 39 | 4.8 | 8.7 | 1.0 | 8 | 3600 | 12 | 5 | 9 | 8 | 1 | 0 | 5 | 0 |
| 29 | 34 | 4.9 | 8.3 | 1.2 | 6 | 3000 | 10 | 2 | 1 | 1 | 0 | 0 | 1 | 0 |
| 30 | 36 | 9.5 | 15.8 | 1.7 | 6 | 3600 | 12 | 5 | 6 | 6 | 3 | 0 | 2 | 0 |
| 31 | 32 | 6.1 | 8.4 | 6.9 | - | 2475 | 11 | 0 | 6 | 4 | 1 | 1 | 2 | 0 |
| 32 | 37 | 5.2 | 6.6 | 2.4 | 12 | 3150 | 14 | 11 | 17 | 17 | 1 | 0 | 14 | 0 |
| 33 | 31 | 5.5 | 5.7 | 2.6 | 8 | 3300 | 12 | 2 | 5 | 5 | 1 | 0 | 4 | 0 |
| 34 | 38 | 6.4 | 6.7 | 5.6 | - | 1650 | 11 | 0 | 16 | 6 | 0 | 0 | 6 | 0 |
| 35 | 33 | 8.8 | 8.7 | 2.7 | 13 | 2250 | 10 | 0 | 7 | 6 | 3 | 0 | 3 | 0 |
| 36 | 35 | 6.2 | 6.1 | 2.5 | 10 | 2200 | 10 | 7 | 9 | 4 | 1 | 0 | 3 | 0 |
| 37 | 25 | 9.0 | 8.8 | 2.7 | - | 2025 | 9 | 12 | 11 | 10 | 9 | 0 | 1 | 0 |
| 38 | 30 | 8.1 | 7.7 | 2.4 | 9 | 3000 | 11 | 8 | 16 | 11 | 2 | 0 | 9 | 0 |
| 39 | 36 | 9.1 | 8.6 | 0.9 | 5 | 4200 | 14 | 5 | 10 | 9 | 1 | 0 | 5 | 0 |
| 40 | 32 | 8.4 | 7.6 | 4.8 | - | 2925 | 13 | 8 | 14 | 2 | 0 | 0 | 2 | 0 |
| 41 | 39 | 5.7 | 5.1 | 3.1 | 8 | 3000 | 10 | 11 | 8 | 6 | 1 | 0 | 4 | 0 |
| 42 | 34 | 4.7 | 4.2 | 6.0 | - | 3175 | 12 | 7 | 24 | 24 | 2 | 1 | 17 | 0 |
| 43 | 35 | 4.6 | 4.0 | 1.7 | - | 3300 | 11 | 11 | 15 | 11 | 2 | 0 | 7 | 0 |
| 44 | 31 | 7.1 | 6.1 | 1.3 | - | 2250 | 10 | 5 | 12 | 11 | 4 | 1 | 6 | 0 |
| 45 | 30 | 6.1 | 5.0 | 4.6 | 12 | 1800 | 8 | 14 | 20 | 17 | 9 | 0 | 7 | 0 |
| 46 | 38 | 2.7 | 2.2 | - | 5 | 3000 | 10 | 1 | 13 | 11 | 3 | 0 | 7 | 0 |
| 47 | 36 | 6.8 | 5.4 | 2.5 | 7 | 3000 | 10 | 3 | 6 | 5 | 1 | 1 | 3 | 0 |
| 48 | 39 | 8.3 | 5.9 | 1.8 | 7 | 3000 | 10 | 9 | 10 | 4 | 0 | 0 | 4 | 0 |
| 49 | 39 | 6.2 | 4.2 | - | 6 | 4200 | 14 | 23 | 10 | 7 | 0 | 0 | 5 | 1 |
| 50 | 37 | 11.0 | 7.2 | 0.6 | 8 | 2100 | 7 | 1 | 1 | 1 | 0 | 0 | 1 | 0 |
| 51 | 36 | 10.5 | 6.8 | 1.0 | 6 | 2100 | 7 | 0 | 1 | 0 | NA | NA | NA | NA |
| 52 | 38 | 7.6 | 4.9 | 2.3 | 10 | 2850 | 10 | 4 | 1 | 1 | 1 | 0 | 0 | 0 |
| 53 | 38 | 6.7 | 3.7 | 1.1 | 7 | 3300 | 11 | 5 | 4 | 3 | 1 | 0 | 2 | 0 |
| 54 | 34 | 11.6 | 3.9 | 0.7 | 6 | 3000 | 10 | 6 | 7 | 5 | 3 | 0 | 2 | 0 |
| 55 | 37 | 133.0 | 6.3 | 2.3 | 8 | 2500 | 10 | 6 | 5 | 4 | 2 | 0 | 1 | 1 |
| 56 | 34 | - | - | 1.6 | 3 | 2700 | 12 | 7 | 10 | 7 | 0 | 1 | 6 | 0 |
| 57 | 33 | - | - | 2.8 | 4 | 2400 | 8 | 0 | 13 | 7 | 2 | 0 | 5 | 0 |

FSH: Follicle-stimulating hormone (mIU/ml); LH: Luteinizing hormone (mIU/ml); AMH: Anti-Müllerian hormone (ng/ml); AFC: Antral follicle count; MII: Metaphase II; Nº: Number; NA: Not applicable; 0PN: Zero pronuclei; 1PN: One pronuclei; 2PN: Two Pronuclei; 3PN: Three pronuclei; -: missing.
